# Editorial: DNA methylation in cancer therapy: therapeutic targets and molecular mechanism

**DOI:** 10.3389/fonc.2024.1508214

**Published:** 2024-12-02

**Authors:** Guoyi Yan, Shaofeng Duan, Jinyang Cai

**Affiliations:** ^1^ School of Pharmacy, Xinxiang University, Xinxiang, China; ^2^ School of Pharmacy, Henan University, Kaifeng, China; ^3^ Department of Human & Molecular Genetics, Massey Cancer Center, Virginia Commonwealth University School of Medicine, Richmond, VA, United States

**Keywords:** DNA methylation, cancer, therapeutic targets, molecular mechanism, biomarker

DNA methylation is a fundamental modification characterized by the addition of a methyl group to the cytosine bases of DNA and plays an important role in regulating gene expression, maintaining genome stability, and influencing cellular differentiation. Since the identification of abnormal DNA methylation in primary human tumors, comprehensive research has been conducted and strongly demonstrates that distorted DNA methylation patterns in cancer can lead to the silencing of inhibitory genes and the activation of oncogenes, thereby promoting tumorigenesis, progression, and resistance to treatment. These alterations in the methylation landscape present both challenges and opportunities for cancer treatment: it cannot only serve as hallmark features of cancer for early detection and monitoring treatment response, but also offer unique insights into potential therapeutic targets and new treatment strategies. The exploration of the molecular mechanisms underlying the changes in DNA methylation levels highlights the intricate relationship between DNA methylation, gene regulation, and cancer pathophysiology, allowing researchers to identify potential therapeutic targets that can effectively and safely inhibit the growth of cancer cells. This Research Topic aims to explore the complexity of DNA methylation in cancer therapy, highlighting its significance as a biomarker for diagnosis and prognosis, as well as a promising avenue for innovative treatment strategies.

Luo et al., attempted to develop a prognostic model for hepatocellular carcinoma (HCC) based on the expression of DNA methylation-related genes to identify potential therapeutic targets and predict overall survival. The researchers analyzed the transcriptomic, clinical, and DNA methylation data from The Cancer Genome Atlas Liver Hepatocellular Carcinoma database and the GSE54236 liver cancer dataset. They identified three methylation-related differential genes- GLS, MEX3B, and GNA14 -and established a risk score model to predict patient prognosis. The model was validated using an independent dataset, and its accuracy was further confirmed through qRT-PCR and immunohistochemistry. The study concluded that the gene signature could provide reliable predictions for clinical applications and enhance the understanding of the mechanisms behind the occurrence and progression of HCC.

Li et al., investigated the correlation between TERT promoter methylation and its expression levels in papillary thyroid cancer (PTC). By analyzing 571 PTC samples from The Cancer Genome Atlas (TCGA), the researchers found that patients with TERT hypermethylation had significantly higher expression level and poorer clinical outcomes compared to those with TERT hypomethylation. Additionally, the study demonstrated that treatment with the demethylating agent decitabine reduced TERT expression and cell viability in PTC cell lines. The findings suggest that TERT promoter methylation can serve as a prognostic marker for PTC risk stratification. The research enhances our understanding of the epigenetic mechanisms underlying the progression of PTC and provides a potential target for therapeutic intervention.

Zhao et al., explored the role of DNA methylation in hepatocellular carcinoma (HCC), focusing on how methylation modifications influence the tumor microenvironment (TME) and the efficacy of immunotherapy. Through a comprehensive analysis of DNA methylation regulators and TME profiles in HCC, the researchers identified three distinct methylation patterns, each with unique TME characteristics. They developed a DNA methylation score (DMscore) to evaluate individual methylation levels, which could predict patient subtype, TME infiltration, and prognosis. A low DMscore, indicative of an inflamed TME with high tumor mutation burden (TMB) and viral infection, was associated with poor survival outcomes. This study suggests that the DMscore can serve as a valuable biomarker for survival and immunotherapy response in HCC patients, potentially aiding in the optimization of immunotherapy approaches.

Ma et al., investigates the association between MGMT promoter methylation, CD47, and TIGIT expression levels and their impact on patient prognosis in adult diffuse gliomas (ADG). This study reclassified ADG into astrocytoma, oligodendroglioma, and glioblastoma (GBM) based on the 2021 WHO classification system. The findings revealed that higher CD47 and TIGIT expression levels in tumor tissues are associated with poorer overall survival rates. Notably, MGMT unmethylation is linked to a poor prognosis in astrocytoma patients. The study suggests that patients with elevated CD47 and TIGIT expression may benefit from anti-CD47 and TIGIT immunotherapy, highlighting the potential of these markers for therapeutic intervention in ADG. However, due to the unbalanced sample size of ADG subtypes and the lack of treated patients, this study acknowledges limitations which precludes definitive conclusions regarding the efficacy of immunotherapy.

In conclusion, this Research Topic of articles highlights the critical role of DNA methylation in cancer development. These findings emphasize the potential of DNA methylation as a valuable biomarker for diagnosis, prognosis, and treatment strategies in oncology.

